# Fruits are vehicles of drug-resistant pathogenic *Candida tropicalis*

**DOI:** 10.1128/spectrum.01471-23

**Published:** 2023-10-31

**Authors:** Yin-Zhi Chen, Kuo-Yun Tseng, Si-Chong Wang, Ciao-Lin Huang, Chih-Chao Lin, Zi-Li Zhou, De-Jiun Tsai, Chiao-Mei Lin, Yu-Lian Chen, Kai-Ting Chen, Yu-Chieh Liao, Feng-Jui Chen, Huey-Kang Sytwu, Chung-Yu Lan, Hsiu-Jung Lo

**Affiliations:** 1 National Institute of Infectious Diseases and Vaccinology, National Health Research Institutes, Miaoli County, Taiwan; 2 Institute of Molecular and Cellular Biology, National Tsing Hua University, Hsinchu, Taiwan; 3 Institute of Molecular Medicine and Bioengineering, National Yang Ming Chiao Tung University, Hsinchu, Taiwan; 4 Institute of Population Health Sciences, National Health Research Institutes, Miaoli, Taiwan; 5 Institute of Molecular and Cellular Biology, National Tsing Hua University, Hsinchu, Taiwan; 6 Department of Life Science, National Tsing Hua University, Hsinchu, Taiwan; 7 School of Dentistry, China Medical University, Taichung, Taiwan; Mayo Foundation for Medical Education and Research, Rochester, Minnesota, USA

**Keywords:** *Candida tropicalis*, fruit, environment, drug-resistance

## Abstract

*Candida tropicalis* is the leading cause of non-albicans candidemia in tropical/sub-tropical areas. We detected a predominant genotype of azole-resistant *C. tropicalis* clinical strains in Taiwan from 2014 to 2018. To investigate the potential fomite transmission of drug-resistant *C. tropicalis*, we characterized yeasts recovered from 17 samples sourced from 6 different kinds of fruits from a supermarket in northern Taiwan in the present study. Fruits were gently washed with buffer, and the solution was then collected for centrifugation. The cell suspension was plated onto CHROMagar Candida medium. Representative yeast species was identified by rDNA sequencing. Drug susceptibilities of *C. tropicalis* were determined by the broth microdilution method. We found that different types of fruit surfaces had different distributions of yeast species. Washing fruit can significantly eliminate the number of yeasts from the surface. Of 123 identified isolates, *C. tropicalis* was the most frequently found species, followed by *Meyerozyma caribbica* and *Candida krusei*. Among 10 collected *C. tropicalis*, all 3 fluconazole-resistant ones were non-susceptible to voriconazole. Furthermore, the genotype of all azole-resistant *C. tropicalis* belonged to the same predominant genotype of azole-resistant *C. tropicalis* causing candidemia in patients in Taiwan. Hence, fruit can serve as a vehicle of azole-resistant *C. tropicalis* and other species, especially *C. krusei*, which is intrinsically resistant to fluconazole.

**IMPORTANCE:**

Of 123 identified isolates from the fruit surface, *C. tropicalis* was the most frequently found species, followed by *Meyerozyma caribbica* and *Candida krusei*. All three fluconazole-resistant *C. tropicalis* were non-susceptible to voriconazole and belonged to the same predominant genotype of azole-resistant *C. tropicalis* causing candidemia in patients in Taiwan. Our findings provide evidence that fruit should be washed before eaten not only to remove chemicals but also potential drug-resistant pathogenic microbes, especially for immunocompromised individuals. To keep precious treatment options in patients, we not only continuously implement antimicrobial stewardship in hospitals but also reducing/stopping the use of agricultural fungicide classes used in human medicine.

## INTRODUCTION

The prevalence of fungal infections has increased significantly in recent decades due to the increase of at-risk populations. Fungal infections contribute to approximately 1·5 million deaths annually (1, 2). Additionally, nosocomial infections also result in extra costs, not only from prolongation of hospital stays but also from other medical costs (3). *Candida* species are the most common pathogens causing severe infections in immunocompromised patients. *Candida tropicalis* has been reported as the most common non-*albicans Candida* species causing invasive candidiasis in tropical/subtropical Asia and Latin America (4 – 10).

Previously, we found that approximately 17% of patients were infected by fluconazole-non-susceptible *C. tropicalis* causing candidemia with cross resistance to itraconazole, voriconazole, and posaconazole, while 55·2% of patients were azole naive (4). Importantly, fluconazole-non-susceptible isolates were genetically closely related, but we did not see time- or place-clustering (4). Furthermore, we also detected that approximately 91% (51/56) of fluconazole-resistant *C. tropicalis* collected from a national survey, the Taiwan Surveillance of Antimicrobial Resistance of Yeasts (TSARY), in 2014 and 2018 belonged to the clade 4 (11), the same genotype causing candidemia detected in our previous non-TSARY hospital study (4). To evaluate the discriminatory power of multilocus sequence typing (MLST), we compared the mitochondrial genome sequence of selected 34 clinical isolates and found that all the clade 4 tested isolates were clustered together (12). Thus, the MLST method is still a convenient and cost-effective tool for studying the genetic relatedness/diversity of *C. tropicalis* isolates. In addition to Taiwan, an increasing prevalence of fluconazole resistance among clinical *C. tropicalis* isolates was reported recently from different areas, including Algeria, China, India, Japan, and Turkey (13 – 19).

In our previous study in 2017, even though one fluconazole-non-susceptible *C. tropicalis* isolate belonging to the clade 4 genotype on a banana surface among 60 fruit samples from supermarkets was detected, no fluconazole-resistant *C. tropicalis* was detected (20). Thus, we conducted a prospective follow-up survey of pathogenic yeasts on the surfaces of fruits to identify potential sources of the predominant genotype of azole-resistant *C. tropicalis* causing infections in humans.

## RESULTS

### Distribution of yeast species

A total of 123 yeast isolates (Table 1 and Table S2) were recovered and identified to species. There were 31, 24, 21, 21, 14, and 12 isolates from pear, mango, lemon, melon, orange, and guava, respectively (see Fig. S1 for representative images of the fruits). A total of 68 pathogenic yeasts composed of 20 species were detected. The four most frequently isolated species that cause infections in humans were *C. tropicalis* (10 instances), *Meyerozyma caribbica* (9 instances), *Candida krusei* (8 instances), and *Moesziomyces aphidis* (6 instances).

**TABLE 1:** Distribution of yeast species isolated from surfaces of fruits^*a*^

Species	Pear	Mango	Lemon	Melon	Orange	Guava	Total
Candida tropicalis*	2	2	1	3	0	2	10
Meyerozyma caribbica*	2	1	3	2	0	1	9
Candida krusei*	3	1	0	3	0	1	8
*Rhodotorula taiwanensis*	1	2	3	0	1	1	8
Moesziomyces aphidis*	1	1	3	0	0	1	6
*Sporidiobolus pararoseus*	2	3	0	1	0	0	6
*Rhodosporidium paludigenum*	0	2	1	2	0	0	5
Kodamaea ohmeri*	0	1	0	2	1	0	4
Kurtzmaniella quercitrusa*	3	0	0	0	1	0	4
Rhodotorula mucilaginosa*	0	0	2	0	2	0	4
*Moesziomyces antarcticus*	0	1	0	3	0	0	4
*Papiliotrema ruineniae*	0	2	0	0	0	2	4
Hanseniaspora opuntiae*	1	0	0	1	0	1	3
*Hyphopichia burtonii*	1	1	0	0	1	0	3
Aureobasidium melanogenum*	0	0	2	0	0	0	2
Candida famata var. famata*	1	0	0	1	0	0	2
Candida metapsilosis*	0	1	1	0	0	0	2
Metschnikowia pulcherrima*	1	0	0	0	1	0	2
Meyerozyma guilliermondii*	1	0	0	0	1	0	2
Trichosporon asahii*	0	0	1	1	0	0	2
Wickerhamomyces anomalus*	1	0	0	0	1	0	2
Yarrowia lipolytica*	0	0	0	0	2	0	2
*Hanseniaspora guilliermondii*	1	0	0	0	0	1	2
*Hanseniaspora pseudoguilliermondii*	0	0	0	0	1	1	2
*Pichia kluyveri*	2	0	0	0	0	0	2
*Sarocladium strictum*	1	1	0	0	0	0	2
*Yarrowia deformans*	1	0	0	0	1	0	2
Others	6	5	4	2	1	1	19
Total	31	24	21	21	14	12	123

^*a*^ *, species has been reported to cause infections in human. One each of *Candida sorboxylosa*, *Papiliotrema flavescens, *Pichia manshurica*, *Pichia mexicana*, *Pseudozyma tsukubaensis*, and *Starmerella bacillaris* from pear; one each of *Cyberlindnera xylosilytica*, *Lodderomyces elongisporus, *Pichia occidentalis*, *Rhodosporidiobolus ruineniae*, and *Rhodotorula toruloides* from mango; one each of *Dirkmeia churashimaensis, *Diutina catenulata, *Hannaella siamensis*, and *Papiliotrema terrestris* from lemon; one of each *Starmerella apicola* and *Torulaspora delbrueckii* from melon; *Hanseniaspora uvarum* from orange and *Wickerhamomyces rabaulensis* from guava.

**Fig 1:**
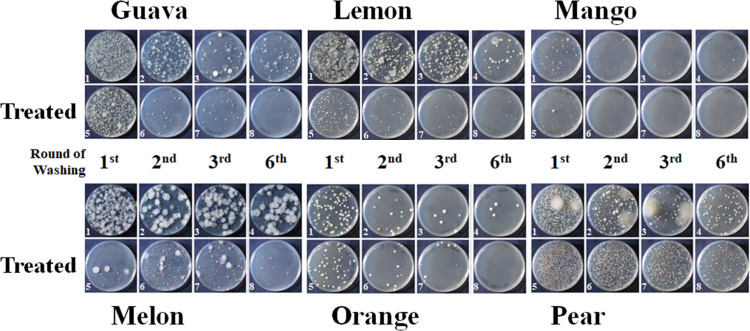
Detection of microbes after treatment. Each sample was washed six times. The colonies on the CHROMagar Candida media of the cell suspensions of the first (1st, 1 and 5), second (2nd, 2 and 6), third (3rd, 3 and 7), and sixth (6th, 4 and 8) washings from whole fruits (1–4) or from fruits of which both ends were removed (treated, 5–8) were photographed after incubation.

**Fig 2:**
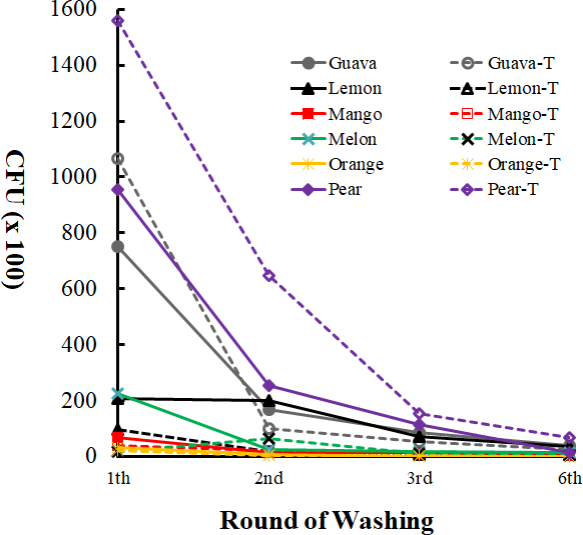
Microbes on the surface of different types of fruit. The effects of washing and removal of both ends were determined. Colony-forming units (CFUs) of 1st, 2nd, 3rd, and 6th washing of whole fruits (solid lines) or from fruits of which both ends were removed (dashed lines) were detected.

We found that different fruits carried different combinations of microbes (Fig. 1). The surface of pear had the highest number of yeasts, followed by guava and lemon. Mango and orange surfaces had a relatively low number of yeasts (Fig. 2). After the third washing, most microbes had been washed off from the fruit surface (Table S3). Removal of the top and bottom ends of lemon, mango, melon, and orange reduced the number of yeast colonies. In contrast, the number of yeast colonies did not decline after the removal of both ends of pear (Fig. 2; Table S3).

### Characteristics of 10 collected *Candida tropicalis* isolates

*Candida tropicalis* was detected on the surface of all fruits other than oranges. We found three isolates on melon; two each on guava, mango, and pear; and one on lemon (Table 2). Three *C. tropicalis* isolates were resistant to fluconazole. Two of those were from the melons, belonging to DST506. The third fluconazole-resistant isolate was from pear and belonged to DST1107. Both DST506 and DST1107 belonged to the clade 4, the predominant genotype of the azole-resistant *C. tropicalis* causing infections in Taiwan (11). Interestingly, of the remaining seven fluconazole-susceptible ones, four belonged to clade 3, and one each to clades 1, 4, and 8 (Table 2). All three fluconazole-resistant isolates were non-susceptible to voriconazole, while YF17294 was resistant to voriconazole, and the remaining two were intermediate. In general, all three fluconazole-resistant isolates were less susceptible to azole used in agriculture (Table 2).

**TABLE 2:** Characteristics of 10 *Candida tropicalis* isolates

Isolate	Fruit collected date	Clade	DST	Fluconazole	Voiconazole	Difenoconazole	Miconazole	Tebuconazole	Triadimenol	*Erg11* ORF		*ERG11* ***mRNA***
										132	154	
YF17026	Mango July 04	8	1106	≤0.125	≤0.0156	≤1	0.25	≤1	≤2	YY	SS	1.5
YF17034	Melon July 04	3	766	0.25	0.0313	≤1	0.5	≤1	≤2	YY	SS	1.5
YF17091	Pear July 04	4	1107	32	0.5	2	4	4	>64	YF	SF	8.4
YF17195	Guava Aug 09	3	1108	≤0.125	≤0.0156	≤1	0.25	≤1	≤2	YY	SS	0.4
YF17229	Melon Aug 08	4	506	8	0.25	≤1	2	≤1	32	YF	SF	1.6
YF17294	Melon Aug 16	4	506	32	1	4	4	4	>64	YF	SF	7.6
YF17304	Guava Aug 16	1	765	≤0.125	≤0.0156	≤1	0.25	≤1	≤2	YY	SS	0.8
YF17310	Mango Aug 17	3	1303	≤0.125	≤0.0156	≤1	≤0.125	≤1	≤2	YY	SS	1.0
YF17358	Pear Aug 17	4	1110	0.25	≤0.0156	≤1	0.25	≤1	≤2	YY	SS	0.7
YF17390	Lemon Aug 21	3	767	0.25	≤0.0156	≤1	0.25	≤1	≤2	YY	SS	1.5

### Mechanism contributing to azole resistance of *Candida tropicalis*

Interestingly, among the three fluconazole-resistant isolates, YF17091 and YF17294 were more resistant to azoles than YF17229 (Table 2). Previously, we detected that a combination of mutation and overexpression of Erg11p, the azole target, was the major mechanism contributing to resistance of clinical isolates (11). To investigate the mechanisms contributing to drug resistance and different levels of azole resistance in the three isolates collected in the present study, we analyzed the Erg11p sequence and *ERG11* mRNA expression of the 10 isolates. All three azole-resistant isolates in the present study had both Y132F and S154F mutations on the Erg11p, consistent with that of isolates from patients (11). Furthermore, the relative levels of *ERG11* mRNA in YF17091 (8.4) and YF17294 (7.6) were significantly higher than YF17229 (1.6) and the remaining eight (ranging from 0.4 to 1.5) fluconazole susceptible ones.

## DISCUSSION

Our findings point to a challenge of azole-resistant *C. tropicalis* in clinical settings. More importantly, the phenomenon of azole resistance in *C. tropicalis* has been observed beyond just Taiwan (21 – 23). Approximately 80% of fluconazole-resistant *C. tropicalis* in China also belonged to the clade 4 (24). The burden of antifungal-resistant *C. tropicalis* in animals or environments is less investigated than it is in humans. Four percent to seventy-five percent of *C. tropicalis* recovered from animals in Brazil, India, and Japan were resistant to fluconazole (17, 25 – 28). Even though limited information was available regarding the rate of azole resistance of *C. tropicalis* recovered from environments, 21% and 25% of isolates from China and Brazil, respectively, were resistant to fluconazole (29, 30). In the present study, we demonstrate that fruit may be one of the sources of fluconazole-resistant *C. tropicalis*, providing the basis for further understanding of the dissemination of drug-resistant pathogenic yeasts.

Selection pressure from the use of clinical azole drugs and/or from patients acquiring azole-resistant *C. tropicalis* from the environment are two major routes for patients with azole-resistant *C. tropicalis*. In our previous studies (4, 11). we found no evidence of patient-to-patient transmission. Nevertheless, there is a possibility that unknown human source is involved, such as that has been reported for *C. parapsilosis* (31 – 34) and multidrug-resistant *Candida auris* (35). Hence, azole-resistant *C. tropicalis* may colonize humans through direct contact or dietary intake of food and water.

The fact that microbes were significantly washed off strongly suggests that for individuals at risk, all kinds of fruits should be carefully washed in running water before being eaten. Also, the fact that fluconazole-resistant *C*. *tropicalis* and *C. krusei* were found, which are intrinsically resistant to fluconazole (36), on fruit surfaces is a reminder of the importance of caution when providing fruits to immunocompromised patients, because fruit surfaces can carry not only pathogenic microbes but also drug-resistant ones.

Since we have found that overexpression of mutated *ERG11*, instead of *CDR1* or *MDR1*, is a major mechanism contributing to fluconazole resistance in clade 4 *C*. *tropicalis* from patients (11), we investigated the potential factors affecting the drug susceptibility for those four clade 4 strains collected in the present study. The YF17358 has wild-type Erg11p and does not overexpress *ERG11* and is susceptible to fluconazole. We found that strains YF17091 and YF17294, which overexpress mutant Erg11p, have a higher level of fluconazole resistance than the strain YF17229, which contains a mutant and does not overexpress Erg11p. This observation is consistent with the stepwise evolution of drug-resistance. Furthermore, whether alternation of *ERG11* gene copy and/or gain-of-function in Upc2p, a regulator of *ERG11*, contributes to the upregulation of *ERG11* needs further investigation. The observation that strains YF17091 and YF17294, which overexpress mutant Erg11p, have a higher level of fluconazole resistance than the strain YF17229, which contains a mutant and does not overexpress Erg11p, is consistent with the stepwise evolution of drug-resistance (37). Furthermore, whether alternation of *ERG11* gene copy and/or gain-of-function in Upc2, a regulator of *ERG11*, contributes to the upregulation of *ERG11* needs further investigation.

Previously, we detected pathogenic yeasts on the fruit surface (20). In addition to patients, we have identified the clade 4 predominant genotype azole-resistant *C. tropicalis* from fruits in the present study. There are limitations in the present study. First, we can’t distinguish whether the clade 4 predominant genotype azole-resistant *C. tropicalis* originated from patients and/or the environment; Second, we cannot distinguish whether the azole-resistant *C. tropicalis* originated from the environment, or farmers of orchards, or was contaminated by customs or supermarket staff. Nevertheless, our findings clearly indicate that fruit can be a vehicle of azole-resistant *C. tropicalis*. In the present study, 17 samples taken from 6 different kinds of available and popular fruits from a supermarket in 3 time periods were analyzed. Increase the number of fruits from various sources and regions or fruits directly from orchards would be our next follow-up investigation to identify a potential hotspot for fluconazole-resistant *C. tropicalis*, but it is beyond the current scope of the study. Third, we did not confirm the genetic relatedness by whole genome sequencing. Depending on different principles, a wide variety of genotyping methods from the traditional pulsed-field gel electrophoresis to the whole genome sequencing are available. Both microsatellite typing and MLST are rapid, specific, and relative highly discriminatory and frequently used to type *Candida* species (38 – 40). In the present study, we used MLST and the data were consistent with those in mitochondrial DNA sequencing (12). One report shows that results from both MLST and microsatellite typing are comparable for *C. tropicalis* (41). However, for some *Candida* species, such as *C. albicans*, *C. parapsilosis*, and *Diutina (Candida) catenulata*, other approaches, such as microsatellite typing, may be more suitable (38 – 40).

How the original clade 4 azole-resistant *C. tropicalis* was selected, either from patients taking clinical azole drugs and/or originating from environments with agricultural fungicides, needs to be investigated. Based on the concept of “one health” (42), antifungal stewardship efforts in hospitals are expected to contribute to a reduction in selection for resistant organisms, but if no parallel efforts are made in agriculture to reduce/stop the use of fungicide classes used in medicine, vulnerable patients would continue to be infected with highly resistant organisms with limited treatment options. Hence, how to implement proper use of azole fungicide in agriculture is equally critical and urgently needed. Furthermore, it is also important to closely monitor the drug susceptibilities of *C. tropicalis*, including clade 4 and non-clade 4, from both patients and environmental sources.

## MATERIALS AND METHODS

### Yeast isolation

To avoid contamination by researchers, we collected fruits carefully to avoid touching them by hand. Then, those fruits were washed in a sterilized bag. Yeasts recovered from 17 samples taken from 6 different kinds of available and popular fruits in 3 different time periods from a supermarket in northern Taiwan in 2017 were characterized. Together, whole fruits from the same sampling were gently washed with 200 mL buffer (1% peptone, 0.5% NaCl) in a 10 L sterilized bag six times. The solution was then collected for centrifugation. To avoid residue from the previous cleaning, the fruits were transferred into a new bag for each round of washing. The pellet was re-suspended in 0.5 mL yeast extract peptone dextrose (YPD) broth. An inoculation loop was used to transfer the cell suspension for plating onto CHROMagar Candida medium (BBL, Becton Dickinson Cockeysville, MD, USA) containing 0.5 g/L chloramphenicol to eliminate the growth of bacteria. After three days of incubation at 25°C, three (if there were) representative colonies of each morphotype were picked for subsequent workup. One isolate per species per sample was analyzed. A total of 139 yeast isolates were analyzed.

### Yeast identification

For identification, all isolates were subjected to ribosomal DNA (rDNA) sequencing. The internal transcribed spacer (ITS) region was amplified by the primers ITS1 5′-TCCGTAGGTGAACCTGCGG-3′ and ITS4 5′-TCCTCCGCTTATTGATATGC-3′, and/or the D1/D2 region of rDNA was amplified by the primers NL1 5′-GCATATCAATAAGCGGAGGAAAAG-3′ and NL4 5′-GGTCCGTGTTTCAAGACGG-3′ (43). Among 139 analyzed isolates, 123 were identified to species. Thus, we reported the characteristics of those 123 isolates.

### Drug susceptibility testing

The drug susceptibilities of *C. tropicalis* were determined by the *in vitro* antifungal susceptibility testing procedure established in our laboratory (10), modified from the guidelines of the Clinical and Laboratory Standards Institute (CLSI) document M27-A3 (44). The growth of each isolate was measured by Multiskan FC Microplate Photometer (Thermo Fisher Scientific, USA) after incubation at 35°C for 24 h.

Standard powders of two clinical antifungal agents, fluconazole and voriconazole, were kindly provided by Pfizer; and azoles used in agriculture—difenoconazol (36531, Merck), miconazole nitrate salt (M3512, Merck), tebuconazole (N-12006, Chem Service), and triadimenol (N-11129, Chem Service)—were dissolved in dimethyl sulfoxide. The final concentrations of difenoconazol and tebuconazole ranged from 1 mg/L to 32 mg/L, 0.125 mg/L to 64 mg/L for fluconazole, 0.125 mg/L to 4 mg/L for miconazole, 2 mg/L to 64 mg/L for triadimenol, and 0.0156 mg/L to 8 mg/L for voriconazole. RPMI medium 1640 (31800-022, Gibco BRL) was used for the dilution and growth of the yeast culture. Strains from American Type Culture Collection (ATCC)—including *Candida albicans* (ATCC 90028), *Candida krusei* (ATCC 6258), and *Candida parapsilosis* (ATCC 22019)—were used as the standard controls.

Minimum inhibitory concentrations (MICs) were defined as the concentration of drugs capable of reducing the turbidity of cells to more than 50%. The newly defined species-specific breakpoints for common *Candida* species were applied in the present study (45). The CLSI newly defined species-specific breakpoints were also applied (46). For fluconazole, the clinical breakpoints were MICs ≤2 mg/L, susceptible; 4 mg/L, susceptible-dose dependent; ≥8 mg/L, resistant. For voriconazole, the clinical breakpoints were MICs ≤0.125 mg/L, susceptible; 0.25 mg/L–0.5 mg/L, intermediate; and ≥1 mg/L, resistant.

### Qualitative analysis of the transcript level of *ERG11* involved in drug resistance by real-time PCR

We further determined the expression levels of *ERG11*. The cells were harvested at OD_600_ 0.7 to 0.9 after growing in YPD liquid medium at 30°C for 6 h. Total RNAs were isolated using the RNeasy Mini kit (QIAGEN), followed by treatment with RQ1 RNase-Free DNase (Promega) to digest the possible contaminating DNA. The purified RNAs were converted to first-strand cDNA using a GoScript Reverse Transcription System kit (Promega). *ACT1* was used as an internal control. The reaction system comprised 10 µL SensiFAST SUBR Lo-ROX kit (meridian), 1 µM forward and reverse primers and cDNAs, in a final reaction volume of 20 µL. Negative controls (DNA-free water) were included in each run. All the real-time PCRs (RT-PCRs) were performed on the QuantStudio 6 Flex (Applied Biosystems). Fold changes in gene expression were determined using the ΔCT method, and data were normalized against the expression of *ACT1*. Then the mRNA level of a fluconazole-susceptible isolate, YFA120877, was used as the denominator for normalization. The primers used in the present study are listed in Table S1.

### Multilocus sequence typing of *Candida tropicalis*

Multilocus sequence typing was conducted as described in our previous report (11, 47). Phylogenetic analysis was performed using the unweighted pair group method with arithmetic average (UPGMA), which was created by MEGA X software. A cutoff *P* distance of 0.01 was chosen because it separated clades that contained known examples of isolates. We constructed a diploid sequence type (DST)-based phylogenetic tree global *C. tropicalis* composed of 1368 DSTs as listed in the *C. tropicalis* MLST database, and clades containing more than 10 genetically closely related DSTs were labeled. The primers used are listed in Table S1.

## SUPPLEMENTAL MATERIAL

Fig. S1 spectrum.01471-23-s0001.tif Photos of the fruits.

Table S1 spectrum.01471-23-s0002.xls Primers used in the present study.

Table S2 spectrum.01471-23-s0003.xls Sources of the 123 isolates from the fruit surfaces.

Table S3 spectrum.01471-23-s0004.xls Yeast colony-forming units of six different types of fruits.

ASM does not own the copyrights to Supplemental Material that may be linked to, or accessed through, an article. The authors have granted ASM a non-exclusive, world-wide license to publish the Supplemental Material files. Please contact the corresponding author directly for reuse.
