# Supplementary figures and images for: Fruits are vehicles of drug-resistant pathogenic Candida tropicalis
Source: Microbiol Spectr. 2023 Oct 31;11(6):e01471-23. doi: 10.1128/spectrum.01471-23 (PMC10714812; doi:10.1128/spectrum.01471-23)

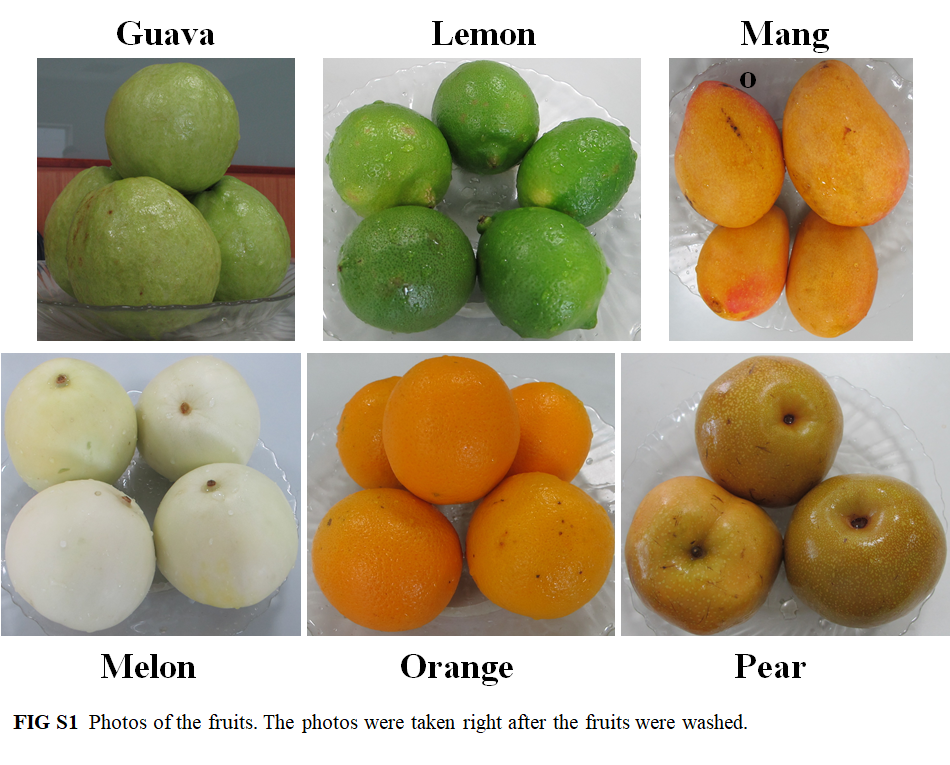

Supplement: Fig. S1 — Photos of the fruits. [file spectrum.01471-23-s0001.tif]
